# Exploring a rare case of juvenile psammomatoid ossifying fibroma in the ethmoid: a case study and review

**DOI:** 10.1093/jscr/rjae242

**Published:** 2024-04-20

**Authors:** Taha Yassine Aaboudech, Hafsa El Ouazzani, Habiba Kadiri, Leila Essakalli, Ayoub Bouteyine, Hanae Benadbdenbi, Naji Rguieg, Nadia Cherradi

**Affiliations:** Pathology Department, Rabat Specialty Hospital, Morocco Mohammed V University in Rabat, Rabat 10100, Morocco; Pathology Department, Rabat Specialty Hospital, Morocco Mohammed V University in Rabat, Rabat 10100, Morocco; Pathology Department, Rabat Specialty Hospital, Morocco Mohammed V University in Rabat, Rabat 10100, Morocco; ENT-HNS Department, Rabat Specialty Hospital, Morocco Mohammed V University in Rabat, Rabat 10100, Morocco; Pathology Department, Rabat Specialty Hospital, Morocco Mohammed V University in Rabat, Rabat 10100, Morocco; Pathology Department, Rabat Specialty Hospital, Morocco Mohammed V University in Rabat, Rabat 10100, Morocco; Pathology Department, Rabat Specialty Hospital, Morocco Mohammed V University in Rabat, Rabat 10100, Morocco; Pathology Department, Rabat Specialty Hospital, Morocco Mohammed V University in Rabat, Rabat 10100, Morocco

**Keywords:** juvenile psammomatoid ossifying fibroma, ossifying fibroma, ethmoid bone, fibro-osseous lesions

## Abstract

Juvenile ossifying fibroma (JOF) and its variants, including juvenile psammomatoid ossifying fibroma (JPOF), represent rare yet clinically significant benign fibro-osseous lesions that primarily occur in children and young adolescents. They can be found in diverse anatomical sites such as the jaw, nasal cavity, paranasal sinuses, and orbit. JOF exhibits an aggressive nature, necessitating early radiological detection and surgical intervention. Similarly, JPOF, with a locally malignant potential, requires surgical removal, typically conducted through endoscopic approaches. We report a case of a 5-year-old girl with JPOF arising in the ethmoid, revealed by recurrent epistaxis and proptosis. The text emphasizes the importance of early diagnosis through histopathology as a diagnostic tool and underscores the need for appropriate management.

## Introduction

Juvenile ossifying fibroma (JOF) is a rare condition primarily observed in children and adolescents. This benign fibro-osseous tumor commonly occurs within the sinonasal region. Its distinctions from the conventional ossifying fibroma lie in the age of onset, anatomical site, locally aggressive behavior, and a notable propensity for recurrence. Clinical manifestations vary based on the tumor’s location, potentially presenting as nasal obstruction, facial swelling, or proptosis. The World Health Organization (WHO) classifies JOF into two main subtypes: juvenile trabecular ossifying fibroma and juvenile psammomatoid ossifying fibroma (JPOF) [1]. The psammomatoid subtype is a rare, benign tumor found in the head and neck region, often affecting children and young adults. It tends to develop in areas such as the nasal cavity, paranasal sinuses, or the orbit. Although typically non-cancerous, it may display locally aggressive behavior, potentially damaging nearby structures. Surgical removal is the preferred treatment, with either endoscopic or external approaches being considered, with a preference for the latter. Due to the risk of significant bleeding during surgery, precautions to secure blood products beforehand are essential [2].

This article outlines a case of JPOF diagnosed in a child.

## Case report

A 5-year-old girl presented with progressive right eye proptosis and recurrent epistaxis over the past 6 months, along with a history of nasal obstruction persisting for several months. During the examination, non-axial proptosis was observed, and both extraocular movements and visual acuity were found to be intact.

**Figure 1:**
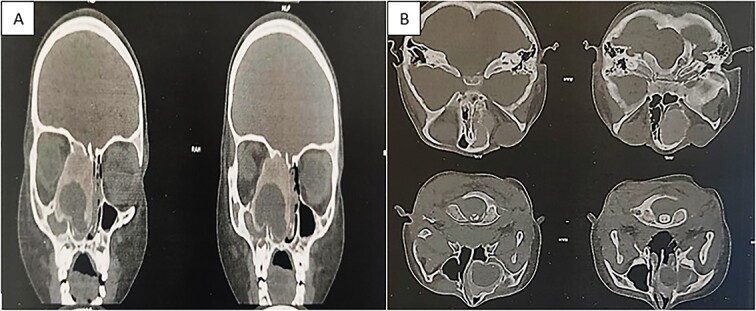
CT showing an expansible, well-demarcated, osteolytic lesion with a peripheral sclerotic rind on the right side of the ethmoid bone, expanding into the ipsilateral nasal cavity, orbit, and maxillary sinus; (A) coronal CT scan; (B) axial CT scan.

Flexible nasofibroscopy revealed a protrusion filling the right nasal fossa, closely approaching the nasal septum. Computed tomography (CT) and magnetic resonance imaging scans indicated a well-defined osteolytic lesion with a peripheral sclerotic rind, measuring 4.1 × 3.8 × 3.7 cm and originating from the right ethmoid, expanding into the ipsilateral nasal cavity, orbit, and maxillary sinus (Fig. 1).

Upon admission to the Ear, Nose, and Throat department, the patient underwent an initial Frozen Section procedure. The sample was promptly sent to the pathology laboratory for histological analysis. The findings indicated the presence of a benign mesenchymal tumor accompanied by roughly round-shaped calcifications. Given the potential for further complications, total tumor removal was deemed necessary to both prevent complications and establish a definitive histological diagnosis.

**Figure 2:**
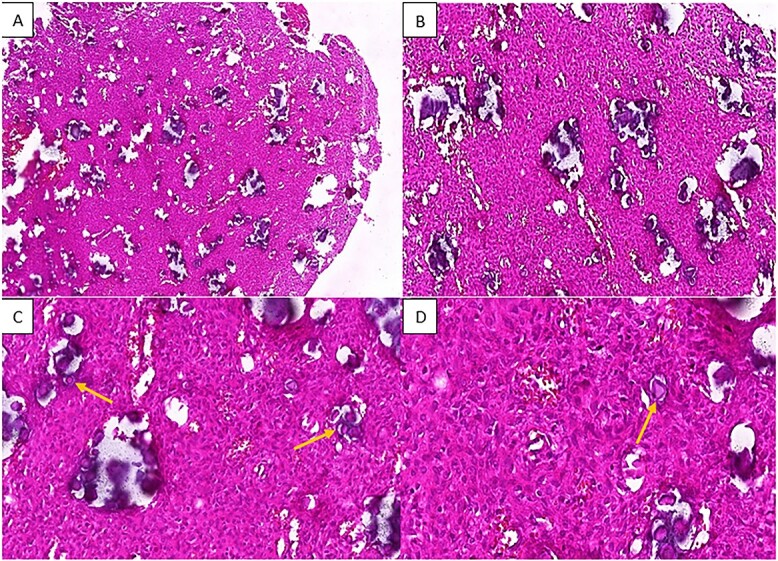
H&E staining of the pathological specimen at different magnifications shows a benign fibro-osseous proliferation characterized by a large number of spherical basophilic calcified entities with no osteoblastic rimming resembling psammoma bodies (arrowheads), within a fibroblastic stroma of uniform stellate or spindle-shaped cells; (A and B) low power magnifications; (C) x20 power magnification; (D) high power (x40) magnification.

The pathology laboratory received a white to reddish fragmented tumor with an elastic to hard consistency. Histological examination disclosed a proliferation of spindle-shaped or ovoid cells with vesicular nuclei and rare mitotic figures. Cells were arranged in sheets, with numerous rounded purplish formations corresponding to psammomatous body-type calcifications. A few giant cells were also observed. The stroma of the tumor was fibrous and collagenous. No necrosis was found (Fig. 2).

**Figure 3:**
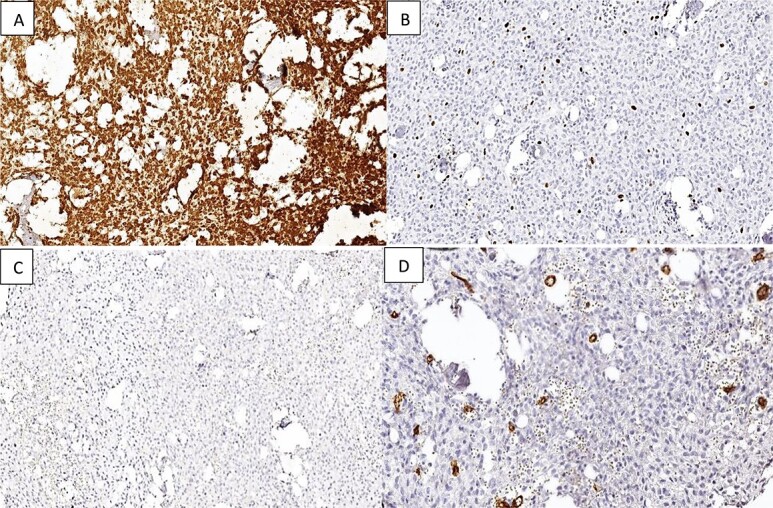
Immunostaining reveals that the tumor tissue is (A) diffusely positive for Vimentin in the stromal component; (B) low Ki-67 proliferation index; (C and D) negative staining for EMA and CD34.

Immunohistochemical studies were conducted, revealing that the tumor cells did not express Cytokeratin, CD34, S100, smooth and striatal muscle markers, MDM2, progesterone receptor (PR), or epithelial membrane antigen (EMA). Vimentin was diffusely expressed, with a very low ki67 proliferation index estimated at 4% (Fig. 3).

## Discussion

This case illustrates a typical clinical history, radiographic appearance, and classic histological findings of JPOF presented in the ethmoid.

JPOF predominantly affects younger individuals, with reported average ages ranging from 16 to 33 years. However, Malek’s review of 86 JPOF cases revealed an age range from 3 to 49 years, with a mean age of 17.7 years and a slight male predominance (1.2:1) [3–5]. This tumor is typically found in the nasal cavity, paranasal sinuses, orbits, or the fronto-ethmoid complex, with an exceedingly rare occurrence in the base or vault of the skull [6]. The primary clinical presentation often involves bony expansion, which is frequently observed. This expansion can manifest through a range of symptoms including proptosis, nasal obstruction, headaches, facial swelling, pain, and recurring sinus infections [7]. Additionally, it may exhibit an aggressive clinical course [8].

Radiologically, ossifying fibroma typically appears as a mass with a distinct border, unlike fibrous dysplasia. Moreover, ossifying fibroma lacks ground-glass opacity and instead exhibits a mixed density resembling both compact bone and fibrous tissue [9].While the appearance of the lesion on imaging might offer diagnostic insights, distinguishing it can be challenging due to its resemblances to other osteo-fibroid lesions within the facial skeleton [10].

Histologically, JPOF is characterized by a large number of spherical calcified entities with no osteoblastic rimming. Some of these calcifications have psammoma-like concentric lamellae with basophilic cores and an eosinophilic osteoid rim. The surrounding fibrous stroma is composed of loosely to densely collagenized tissue that contains a dense proliferation of fibroblast-like, spindle-shaped cells with hyperchromatic nuclei [11].

JPOF must be distinguished from other fibro-osseous lesions such as fibrous dysplasia, osteoblastoma, low-grade central osteosarcoma, and primary aneurysmal bone cyst. Additionally, it is important to consider intraosseous cavernous hemangioma and eosinophilic granuloma due to their similarities in radiological and histological features [6, 12]. Misdiagnosis may occur with extracranial meningioma featuring psammoma bodies, which typically tests positive for EMA, unlike JPOF [7]. JPOF typically does not express S100 and CD34 [7].

Despite its benign and slow growth, this subtype exhibits local malignancy with a tendency to infiltrate surrounding structures. Therefore, accurate diagnosis is crucial, and treatment should involve complete tumor removal. Incomplete or partial resection is associated with a heightened recurrence risk [2].

## Conclusion

Our report underscores the rarity of JPOF, focusing on its clinical, radiological, and primarily histological aspects. Despite its uncommon occurrence, JPOF poses diagnostic and management challenges due to its potential aggressiveness and recurrence. Histopathological examination remains pivotal for confirming the diagnosis, highlighting the importance of thorough evaluation and appropriate management guided by histological findings.

## Conflict of interest statement

None declared.

## Funding

No external funding sources were obtained for this submission.

## Data availability

No new data were generated or analyzed in support of this research.

## Consent for publication

Written informed consent for publication of their clinical details and/or clinical images was obtained from the patient.
